# Interrogation of alternative splicing events in duplicated genes during evolution

**DOI:** 10.1186/1471-2164-12-S3-S16

**Published:** 2011-11-30

**Authors:** Ting-Wen Chen, Timothy H Wu, Wailap V Ng, Wen-Chang Lin

**Affiliations:** 1 Institute of Biomedical Informatics, National Yang-Ming University, Taipei, Taiwan; 2 Institute of Biomedical Sciences, Academia Sinica, Taipei, Taiwan

## Abstract

**Background:**

Gene duplication provides resources for developing novel genes and new functions while retaining the original functions. In addition, alternative splicing could increase the complexity of expression at the transcriptome and proteome level without increasing the number of gene copy in the genome. Duplication and alternative splicing are thought to work together to provide the diverse functions or expression patterns for eukaryotes. Previously, it was believed that duplication and alternative splicing were negatively correlated and probably interchangeable.

**Results:**

We look into the relationship between occurrence of alternative splicing and duplication at different time after duplication events. We found duplication and alternative splicing were indeed inversely correlated if only recently duplicated genes were considered, but they became positively correlated when we took those ancient duplications into account. Specifically, for slightly or moderately duplicated genes with gene families containing 2 - 7 paralogs, genes were more likely to evolve alternative splicing and had on average a greater number of alternative splicing isoforms after long-term evolution compared to singleton genes. On the other hand, those large gene families (contain at least 8 paralogs) had a lower proportion of alternative splicing, and fewer alternative splicing isoforms on average even when ancient duplicated genes were taken into consideration. We also found these duplicated genes having alternative splicing were under tighter evolutionary constraints compared to those having no alternative splicing, and had an enrichment of genes that participate in molecular transducer activities.

**Conclusions:**

We studied the association between occurrences of alternative splicing and gene duplication. Our results implicate that there are key differences in functions and evolutionary constraints among singleton genes or duplicated genes with or without alternative splicing incidences. It implies that the gene duplication and alternative splicing may have different functional significance in the evolution of speciation diversity.

## Background

Gene duplication is one way to increase genome complexity, and may provide a source for genetic novelty. After duplication, genes are temporally released from evolutionary constraint, and may be subject to pseudogenization, subfunctionalization or neofunctionalization [1]. Previous results have demonstrated that after duplication those duplicates had a higher evolutionary rate, due to relaxation of purifying selection [2-5]. This acceleration of evolutionary rate may create divergent function(s) for one or both of the duplicates. Gene duplication provides raw material for generating new protein function, and accordingly, not all gene duplication events could be preserved and eventually fixed in the population. It has been shown in yeast genomes that complex genes with longer protein sequences, more protein domains and more cis-regulatory regions are more likely to remain as duplicated genes after a whole genome duplication event [6,7].

In addition to duplication events, some genes in eukaryotes can undergo alternative splicing to increase their expressional flexibility, and increase the complexity at the transcriptome or proteome level [8,9]. The relationship between alternative splicing (AS) and gene duplication is an interesting question in the evaluations of gene functions and evolution. Previous studies have shown that there was a negative correlation between gene duplication events and AS especially in newly duplicated genes in human [6,10], and *C. elegans*[11,12]. These results implied that duplication and AS may share similar function and therefore can be interchangeable in evolution [10,13]. However, there was also a recent study reported where duplicated genes actually had a higher proportion of AS and larger number of AS isoforms per duplicated locus in human [14]. Whether duplicated genes tend to gain more AS compared to single copy genes (singletons), and the relationship between gene duplication and AS in evolution, are unsolved and interesting questions to be answered.

There are two advances we make to help answer this question. First, it has been found that fewer newly duplicated genes possess AS, and it also has been suggested that the acquirement of AS after gene duplication is one factor to avoid pseudonization or gene loss in plants and *Drosophila *[15,16]. Therefore, the age of duplication may play an important role in acquiring AS. Hence we took this into consideration in this study by defining gene duplicates with different identity criteria. Second, aided by improved sequencing technology and increasing EST and cDNA experimental data, many more alternative splice isoforms had been identified [17,18]. Most human genes are expected to have alternative splicing isoforms [19]. The relationship between alternative splicing and gene duplication using the newly updated data is worthy of further investigation.

In addition to the relationship between duplication and AS, since these are two different means to achieve diversity, we think that genes which employ both, either, or none of these two separate strategies might be distinct from each other in terms of evolutionary rates, and in other characteristics such as functions. Therefore, we study the length of protein product, number of domains, evolutionary rate, and gene functions for these four groups (duplicates with/without AS and singletons with/without AS) of genes.

## Results

### Duplicates acquired more AS than singletons

**Figure 1:**
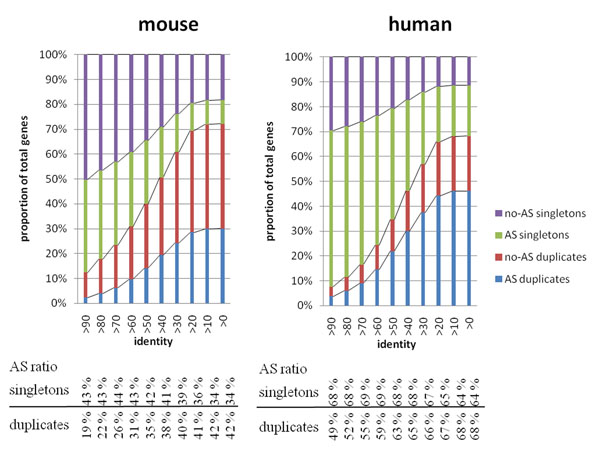
**Distribution of singletons and duplicated genes with and/or without AS.** This figure shows the distribution of singleton and duplicates across different criteria of percent identity. The ratios of singletons (or duplicates) having AS under each criterion are also listed at the lower panel of the figure. For those high identity criteria groups, only recently duplicated genes are considered as duplicates, while for those lower identity criteria, both new and anciently duplicated genes are grouped as duplicates. As the percent identity threshold for a duplicated gene becomes lower, the proportion of duplicated genes (summation of red and blue) grows. The proportion of duplicated genes having alternative splicing also grows, and the growth rate is actually faster than the growth rate of duplicated genes. As a result, the proportion of genes having AS among duplicates is statistically significantly greater than the proportion of AS among singletons at lower identity criteria (identity >20 for both human and mouse). That is, those ancient duplicated genes have more alternative splice isoforms than singletons.

In order to further investigate the relationship between alternative splicing and gene duplication, we compared the proportion of genes with/without AS and duplication. All genes in human and mouse were divided into four groups: with neither AS nor paralog (no AS singletons), with AS but not paralog (AS singletons), with paralog but not AS (no AS duplicates), and with both AS and paralog (AS duplicates). In order to distinguish those newly duplicated genes and anciently duplicated genes, paralogs were identified with different identity criteria. The distribution of those four groups across different identity criteria for human and mouse is shown in Fig. 1. As one may expect, with a higher identity criterion, only those recently duplicated genes were identified as duplicated paralogs, while with a lower identity criterion, those more ancient of the paralogs were included as duplicated genes. Therefore, the proportion of duplicated genes (summation of AS duplicates and no AS duplicates) increases as the identity criterion decreases. We were interested in the alterations of relative proportion of AS in duplicated genes and singletons.

The proportion of these four groups varied at different identity criteria. Under the high identity criterion, many genes are classified as singletons, and the AS proportion for singletons is much higher than that observed for duplicates. There is a low proportion of AS in duplicates under a high identity criterion, and the proportion of AS increases as the identity criterion becomes lower. Take an identity criterion >90 and >20 in human as an example, 49% and 67% of duplicated genes have AS, respectively. On the other hand, 68% and 65% of singleton genes have AS for identity >90 and >20, respectively. Under an identity criterion >90, there is a significant enrichment of AS in singleton genes (Pearson's Chi-squared test, p-value < 2.2e-16). While under an identity criterion >20, the trend is reversed, and there is a significant enrichment of AS in the duplicated genes (Pearson's Chi-squared test, p-value = 0.01). Given that at a lower identity criterion the gene families identified include those gene families identified at a higher identity criterion, and that AS proportion still increases at a lower identity criterion, we could observe that anciently duplicated genes overall have a much higher proportion of AS compared to those recently duplicated genes and singletons. Similar reasoning can be made in the subsequent analyses.

### Smaller gene families have higher AS proportion and more AS isoforms contrary to large gene families

**Figure 2:**
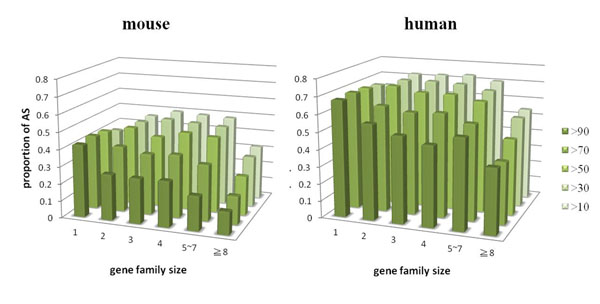
**Proportion of AS**. Proportion of AS in different sizes of gene families and singletons across different identity criteria. Gene families identified by different identity criteria were grouped according to their family size and the proportions of AS were calculated for each group. There is consistently a lower proportion of AS in large gene families across different identity criteria in both human and mouse. This negative correlation between proportion of AS and protein duplication is more significant for those darker green groups. For genes with the same gene family size, the proportion of AS increases as the identity criterion decreases.

In addition to the occurrence of duplication, the number of duplicates in the gene families was shown to be correlated with the AS proportion, and the number of AS isoforms [10,14]. Therefore, we further investigated the relationship between the size of gene families and AS across different identity criteria. We stratified duplicated genes into gene families of different family size, and plotted the AS proportion for different identity criteria (Fig. 2). We found that the AS proportion seemed to decrease in larger-sized gene families at a higher identity criterion. When a lower identity criterion was used, this negative correlation between gene family size and AS proportion became less evident. Meanwhile, we found that there was consistently the lowest AS proportion for large gene families (gene families possessing at least 8 duplicates) across all identity criteria.

It is worth noting in Fig. 2 the trend of increasing AS proportion in duplicated genes as the identity criterion gets lower. The AS proportions of duplicated genes become larger than that of singletons at those lower identity criteria. If we take singletons (gene family size =1) into consideration, the AS proportions of singletons are larger than all sizes of gene families at higher identity criteria (>70 or >90), but become smaller than those small and moderate gene families (gene family size range from 2 - 4 or even 5 - 7) at lower identity criteria (>50, >30 or >10). Even though the AS proportions for large gene families (greater or equal to 8) remain lower than singletons across all identity criteria, the AS proportions for other gene families (gene families size from 2 to 7) are significantly higher than that in singleton groups at a lower identity criterion, especially for the group identity >10. This result shows that our finding of a higher AS proportion for duplicated genes was mainly contributed by those slight or moderately duplicated gene families.

**Figure 3:**
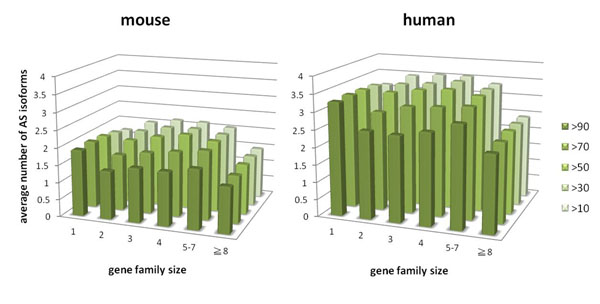
**Average number of AS isoforms.** Average number of AS isoforms for singleton and duplicated genes across different identity criteria. For gene duplicates identified by >90 identity criterion (bars with darkest green) there is a decrease in the number of average AS isoforms compared to the number of average AS isoforms of singleton genes in both human and mouse. On the other hand, for those gene families identified by >30 or >10 identity criteria, the number of average AS isoforms in smaller gene families (≦7) is larger than singletons, which in turn is still larger than the average number in the big gene families (≧8).

In addition to the AS proportion, the average number of AS isoforms was also previously shown to be correlated with the size of gene families. It was shown that there were on average fewer AS isoforms in large gene families in both human and *C. elegans *[10,11]. In order to investigate whether this phenomenon existed in our dataset, we calculated the average number of AS isoforms for different gene family sizes across different identity criteria as shown in Fig. 3. The big gene families (gene families having at least 8 paralogs) do have fewer AS isoforms in both human and mouse at all identity criteria. There are fewer AS isoform numbers in all sizes of gene families at higher identity criteria (>90 or >70). This is consistent with previous findings that the newly duplicated genes usually do not have AS [15]. On the other hand, at those lower identity criteria (>50, >30 or >10), the AS isoform numbers of those small and moderate gene families (gene family size no more than 7) increase and become larger than comparable singletons. It suggests that those anciently duplicated genes tend to evolve toward having more AS isoforms compared to those non-duplicated genes. Interestingly, we notice that there is a slight rise in the number of isoforms for those moderately sized gene families (gene family size 5 - 7) for both human and mouse, even at higher identity criteria (>90 and >70).

### Features comparison among AS/no AS duplicates/singletons

The positive correlation between duplication and AS found at lower identity criteria indicates that there are genes that somehow tend to both gain duplication and undergo AS, while there are other genes that have neither in the long-term consideration. This suggests that there may be two extreme types of genes, one is more likely to become diverse whether through gene duplication or AS. The other type does not need to become diverse, and tends to remain as a single copy gene in the genome, and may also express only one transcript. In addition to these two extreme types of genes, there are still some genes which may gain either AS or duplication. We are interested in the differences in features between these four groups of genes which have not been examined in previous studies. In order to investigate these questions, we make comparisons among the following four groups: gene family where all of the members have AS (A_F), gene family where none of the members of the family has AS (N_F), AS singletons (A_S) and no AS singletons (N_S). We investigated the difference in protein length, domain number, evolutionary rate, and GO distribution in these four groups and found some significant and evident differences did exist.

### Length of protein product

**Figure 4:**
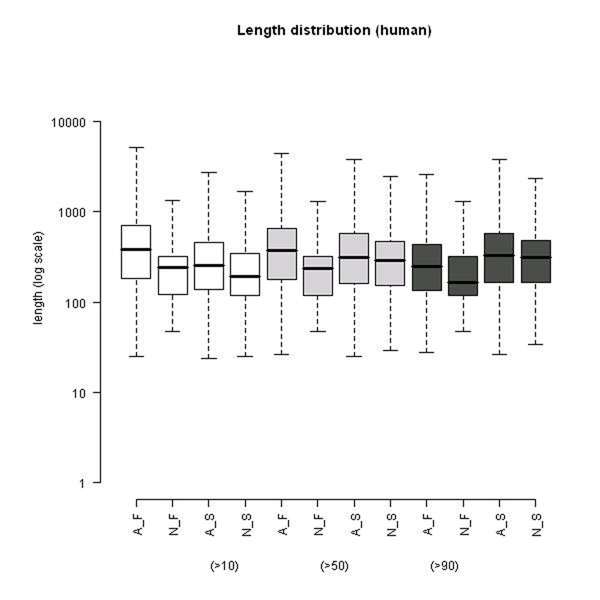
**Box plot of protein lengths.** Protein length of four groups of genes: A_F, N_F, A_S, and N_S (for AS gene families genes, no AS gene families genes, AS singletons, no AS singletons, respectively) of human across three different identity criteria (>10, >50, >90). When comparing within the same identity and the same duplication status, the length of genes with AS are longer than genes without AS, and this tendency is especially obvious for duplicated genes (gene families) or at lower identity criterion (>10).

We plotted the distribution of protein length for all of these four groups of genes from human (Fig. 4). The average length of A_F is longer than N_S for both >10 and >50 identity criteria. We found that overall genes with AS were longer than those without AS. The same phenomena could be observed in mouse data as well (Additional file 1). This kind of difference was most evident for those duplicated gene families. We also found that the most recently duplicated genes without AS seems to be the shortest group. In a recent study, tandem exon duplications and following alternative splicing had been shown to increase the diversity of metazoans [20]. We propose that after gene duplication, some genes may have evolved to become longer (e.g. by exon duplication) and then gained AS, or they may have evolved to gain AS then became longer.

### Distribution of number of domains

**Figure 5:**
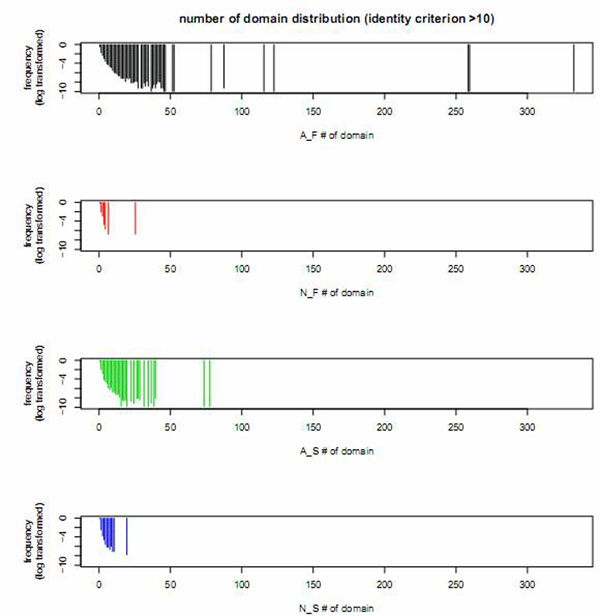
**Number of domain(s)**. Domain number distribution for genes of four groups of genes: A_F, N_F, A_S, and N_S (for AS gene families genes, no AS gene families genes, AS singletons, no AS singletons, respectively) identified with identity criterion >10. The y axis is a log transformed frequency among each group. The lower the frequency, the longer the line is on the plot, and the higher the frequency the shorter the line is. There are sparse lines for domain number larger than 50. For both gene families and singletons the no AS genes have less number of domain(s) compared to AS genes. Overall, N_F have the least number of domains.

We also analyzed the distribution of these four groups of human genes based on number of domains (Fig. 5 for identity >10, and Additional file 2 for identity >90 and identity >50). As shown on the plots, the rankings of the groups by the number of domains are different under different identity criteria. Nevertheless, genes without AS always have fewer number of domains whether they are duplicated or not. In addition, the N_F have the least number of domains across all identity criteria. On the other hand, for identity criterion >10, A_F have the greatest number of domains followed by A_S, N_S, and finally N_F. For identity criteria >50 or >90, the group with the largest number of domains became A_S (Additional file 2). This can be explained as those genes containing large number of domains are more likely to be grouped into duplicated genes while using lower identity criterion (e.g. >10) compared to genes containing fewer domains. That is, those anciently duplicated genes with AS tend to be genes having many more domains compared to the background. Besides these gene features, the evolutionary rate after gene duplication is also an interesting topic. We further investigated the evolutionary rates of those four groups.

### Evolutionary rate

**Figure 6:**
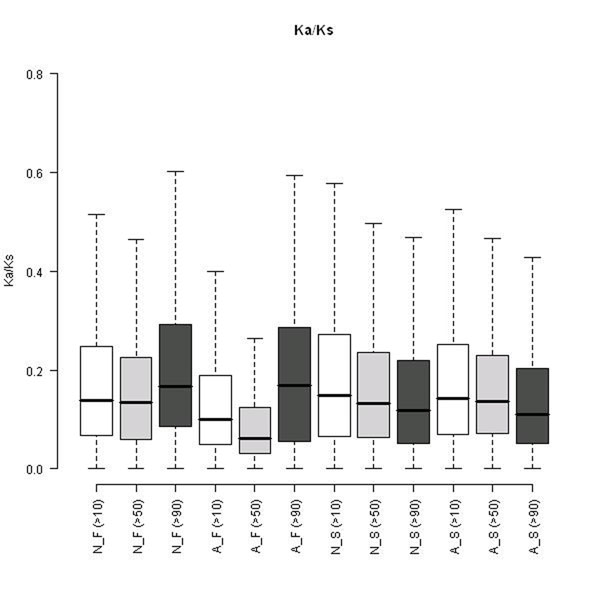
**Boxplot of Ka over Ks.** Boxplot of Ka/Ks value for the four groups: N_F, A_F, N_S, and A_S (no AS gene families genes, AS gene families genes, no AS singletons, and AS singletons, respectively) across three different identity criteria (>10, >50, and >90). Overall, there is no significant difference observed for Ka over Ks values among singletons. For duplicated gene sets, the most similar gene families N_F (>90) and A_F (>90) have the top two highest Ka over Ks which imply a higher evolutionary rate. Conversely, those more anciently duplicated genes with AS, i.e. A_F (>10) and A_F (>50), have the lowest Ka over Ks values.

The evolutionary rate of the four groups of genes across different identity criteria was analyzed. The Ka and Ks of human genes derived from comparison of mouse orthologs were used to calculate the Ka over Ks values and the results were plotted and are shown in Fig. 6. We found that these recently duplicated genes (gene families identified by >90 identity criterion) had significantly higher Ka over Ks. For the case under identity >50 and >10, even though the N_F seem to have similar Ka over Ks value with singletons, the overall Ka over Ks value is higher for singletons compared to those duplicated gene sets (for the total gene number, A_F are much more than N_F). These results are consistent with previous reports which suggest that the duplicated genes actually are more conserved compared to singleton genes, even though they may have an accelerated evolutionary rate soon after the duplication [21,22]. Interestingly, we found that among those more anciently duplicated genes, gene families without AS had higher evolutionary rates compared to gene families with AS. We obtained similar results when we used the Ka and Ks values derived from human and chimp orthologs instead (data not shown), only that we found the N_F had even slightly higher evolutionary rate compared to singleton genes in that case. As our results indicate, there is a different evolutionary constraint on those no AS duplicates compared to duplicates with AS, the function of those two different groups of genes are interesting and in need of further exploration.

### GO analysis

**Figure 7:**
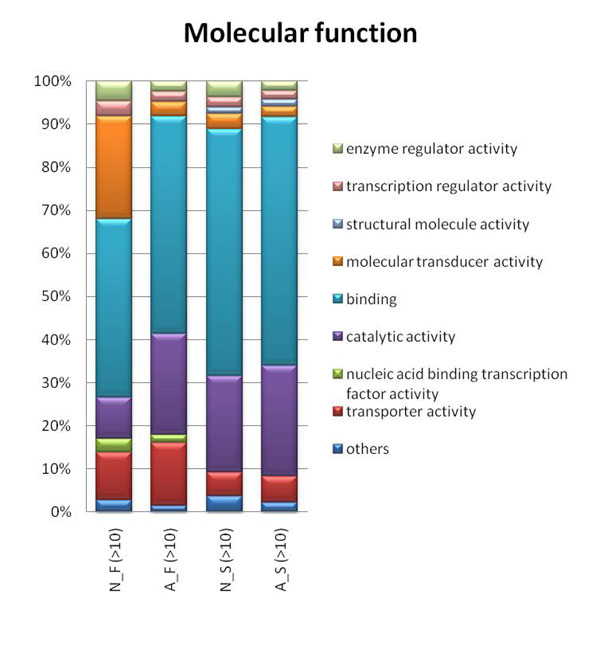
**Molecular function distribution.** Molecular function distribution for four groups of genes: N_F, A_F, N_S, and A_S (no AS gene families genes, AS gene families genes, noAS singletons, and AS singletons, respectively). Only categories having proportion larger than 1% are shown, and categories having a proportion smaller than 1% are tallied together as “others”. There is an enrichment (more than 6 times) of molecular transducer activity for N_F compared to the other three groups. The molecular transducer activity for N_F, A_F, N_S and A_S are 23.9%, 3.3%, 3.6% and 2.3% respectively.

**Figure 8:**
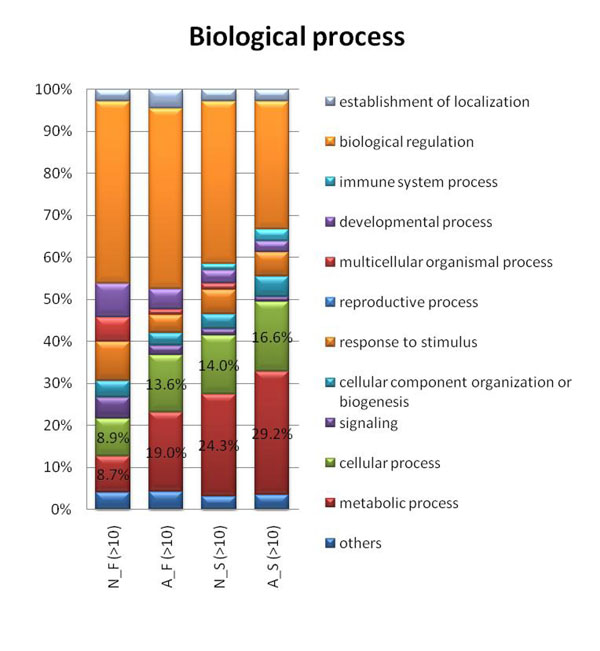
**Biological process distribution.** Biological process distribution for four groups of genes: N_F, A_F, N_S, and A_S (no AS gene families genes, AS gene families genes, no AS singletons, and AS singletons, respectively). The category “others” is derived similarly as in Fig. 7.

We investigated the distribution of molecular functions and biological processes for the four groups of genes. For molecular function, there is an apparent enhancement of “molecular transducer activity” for N_F (Fig. 7). In addition, the “nucleic acid binding transcription factor activity” is slightly enhanced for duplicated genes (for both N_F and A_F) and the “structural molecule activity” is enriched for singletons. We also found genes without AS are enriched with the ‘enzyme regulator activity’ category comparing to genes with AS for both duplicates and singletons. On the other hand, for “catalytic activity”, singletons with AS are slightly more enriched than singletons without AS, whereas duplicates with AS are much higher than duplicates without it. For biological process, the distribution patterns for the four groups vary by a significant amount (Fig. 8). There is a higher proportion of the “multicellular organismal process” and “signaling” annotations for N_F. Meanwhile the “metabolic process” and “cellular process” annotations are most abundant for A_S followed by N_S, A_F, and N_F groups. Genes possessing AS had previously been shown to have an enrichment in certain molecular functions (immune and nervous systems) [23]. In our results, the immune system process does have about double the proportion for A_S compared to N_S (2.9% compared to 1.5%) but this trend is not found in duplicated genes. For the four groups identified by >50 and >90 identity criteria, the results are similar (data not shown).

## Discussion

Previous studies have suggested that duplication and AS may be interchangeable and therefore were reversely correlated [10,13]. On the other hand, a recent study suggested a positive correlation [14]. The apparent discrepancy may be due to the availability of AS data at the times of these studies, or how the duplicates were defined. Previous studies identified duplicated genes by either InParnoid [24] or BLASTP which are both based on sequence similarity [10,13,14]. In this study, we used duplicates identified by EnsemblCompara GeneTrees which also took the phylogenetic tree structure into consideration and may provide higher coverage and better ability to handle large gene families [25]. The discrepancy may most probably be due to the similarity cutoffs used to determine duplicates, which are reflections on the age of duplications. Therefore in this paper, we investigated the relationship between duplication and AS across different duplication times (e.g. Duplicates were identified according to identity >10, >50 or >90). We found that in human and mouse overall the duplicated genes had higher proportion of alternative splicing after long-term evolution, even though those recently duplicated genes tended not to have AS. This finding is consistent with a recent publication which concluded that the gain of AS in duplicated genes is in a age-dependent manner [26].

The evolutionary rate of genes after gene duplication was though to be increased due to the relaxation of selection [4]. Previously, Jin et al. proposed an “Accelerated AS model”, which suggested that the relaxation of constraint for duplicates after gene duplication may accelerate the rate of AS acquirement [14]. Our results do not directly support nor reject the hypothesis; we could not conclude whether the acceleration exists since the time needed for the acquisition of alternative splicing is unclear. However, we made an interesting observation that although duplicated genes did have a higher evolutionary rate (Ka/Ks), the proportion of AS among recently duplicated genes (identity >90) was lower than those of singleton. It was only after the inclusion of ancient duplicates did the proportion of AS becomes higher compared to that of the singletons.

We did try to investigate whether this trend exists in other mammalian species. We observed the same trend by choosing 4 relatively well-studied mammals: human, mouse, rat, and chimp, and used the “all” information (without removing those peptides labeled as “novel”, and including all paralogous information) provided by Ensembl to perform the same analysis. The fractions of alternative splicing among their singleton gene and duplicated gene sets are listed in Table 1. Even though there is a trend that better-studied species do have higher fraction of alternative splicing in both duplicated gene and non-duplicated gene groups, the average alternative splicing rate in the duplicated genes is still significantly higher than the rate in the non-duplicated genes in all 4 mammalian species. It is likely this phenomenon exists in all mammals.

**Table 1:** Proportion of Alternative splicing (AS) for singletons and duplicated genes of four mammalian genomes.

Organism	AS proportion for singletons	AS proportion for duplicates
human	66.5%	73.2%
mouse	41.0%	52.7%
chimp	34.7%	42.7%
rat	25.6%	31.9%

* paired t test, p value = 0.0035

On the other hand, we did not observe this kind enrichment of AS for duplicated genes in zebrafish, fly, and worm. This may result from different evolutionary stresses between mammalian and other eukaryotes and therefore the preference of alternative splicing and gene duplication in the genome are different. It is also possible that this trend does exist in other eukaryotes but is not being observed only because of the lack of alternative splicing information for zebrafish, fly, and worm. We actually found a tendency to increase in proportion of alternative splicing in duplicated genes in both zebrafish and fly, even though they are not statistically significant. A recent study in *Drosophila* also showed that newly duplicated genes usually did not have alternative splicing isoforms and were expressed at a lower expression level, while those genes may have gained their diverged function or expression patterns after they developed alternative splicing activity [15]. It is possible that we did not observe the same trend in zebrafish, fly, and worm simply due to the lack of proper AS information. As NGS technique improves and data accumulates, further investigation can be done to answer this question in the future.

Genes categorized as having no AS may really have none, but it is also possible that its AS isoforms are not detected due to their relatively low level of expression [27]. In order to examine this possibility in our dataset, we tested whether those genes express at a relatively lower level by exploring current human EST datasets. The result of the EST hit distribution is shown in Additional file 3. We found that there was indeed a high correlation between the EST counts and AS detection. Genes without alternative splicing annotation may be a misleading artifact arising from its low expression level, and it is possible that their AS isoforms are not yet found. Therefore we could not rule out the possibility that a portion of the no AS gene families may actually have AS. However, the apparent lack of AS prompted us to attribute these genes into the no AS categories.

As previous studies on gene retention after whole genome duplication have suggested, subsets of genes were more likely to remain as duplicates and may be more likely to evolve diverse functions [28,29]. Among those genes apt to become diverse, some develop either duplication or AS, and still some gain both duplication and AS. From our results, we suggest that longer genes and genes which contain more domains are more likely to increase their gene diversity at the genomic level via gene duplication, and increase their complexity at the transcriptome level by acquiring alternative splicing. Or in alternative, genes which undergo duplication and alternative splicing may become longer and may increase their number of domains. In addition, we found that there are different evolutionary rates and preferences for molecular function or biological process for no AS gene families and AS gene families. These all imply that the selection between duplication and AS are highly dependent and is not random. Further investigation on what factors affect gene duplication and AS may reveal the evolutionary relationship between those two mechanisms. In conclusion, our analysis revealed a sophisticated relationship between duplication and AS depending on the age of the duplicates. Compared to singletons, there is actually a higher proportion of ancient duplicates that have AS isoforms, while fewer new duplicates have alternative splicing isoforms. In addition, among those duplicated genes, genes with longer sequence and more domains are more likely to develop AS. As for the evolutionary rate, our results suggest that in consideration of a long-term evolution, duplicated genes may be under higher evolutionary constraints compared to singleton genes, and among those duplicated genes, those having AS are the ones actually under higher constraints compared to those having no AS.

## Conclusions

Our analysis revealed a sophisticated relationship between duplication and AS depending on the age of the duplicates. Compared to singletons, there is actually a higher proportion of ancient duplicates that have AS isoforms, while fewer new duplicates have alternative splicing isoforms. In addition, among those duplicated genes, genes with longer sequence and more domains are more likely to develop AS. As for the evolutionary rate, our results suggest that in consideration of a long-term evolution, duplicated genes may be under higher evolutionary constraints compared to singleton genes, and among those duplicated genes, those having AS are the ones actually under higher constraints compared to those having no AS.

## Methods

### Materials

The protein sequences and paralog information of seven species: human (*Homo sapiens*), mouse (*Mus musculus*), rat (*Rattus norvegicus*), chimp (*Pan troglodytes*), zebrafish (*Danio rerio*), worm (*Caenorhabditis elegans*) and fly (*Drosophila melanogaster*) were downloaded from Ensembl release 60 [30]. For most analysis, only the peptides labeled as “known” were preserved. The Ka, Ks, cDNA sequences and protein GO category of human and mouse were also downloaded from Ensembl.

### Duplication and AS identification

The paralog pairs were generated by grouping paralog information derived from EnsemblCompara GeneTrees [25]. Different set of paralog pairs are identified at different identity criterion. A paralog pair is qualified as a pair if at least one gene aligns to the other with a protein sequence identity larger than the identity criterion chosen. Genes were classified as duplicated genes if they have any paralog(s). Gene pairs were clustered together as a gene family if there was at least one gene in common. Gene families were further classified into AS gene families, no AS gene families, and others. AS gene families were defined as gene families within which all paralogs have AS, and no AS gene families were defined as gene families within which none of the paralogs have AS. Genes were classified as having alternative splicing isoforms if their genes have more than one protein product recorded as known peptides in Ensembl.

### Features of genes analysis

The raw data of gene information downloaded from Ensembl were further used in analyzing human (and mouse sometimes) for protein function, domain number, gene length, and Ka over Ks analysis. Domains in protein sequences were identified by RPS-BLAST using pfam release 24.0 as database [31] with default parameters, except the expected value is set be smaller than 0.01.

### EST analysis

Human EST data were downloaded through the NCBI ftp server: http://www.ncbi.nlm.nih.gov/genbank/. Not all EST data were suitable for expression analysis since some libraries may have been subject to expression count distortions such as amplification or normalization. Therefore, EST libraries were subject to manual reviews based on extended annotations of clone libraries obtained from the Cancer Genome Anatomy Project (CGAP) http://cgap.nci.nih.gov/. ESTs from unsuitable libraries were eliminated. Representative transcription isoforms were also selected and repeat-masking was applied with RepeatMasker [32]. ESTs from the prior procedure were aligned against the set of transcription isoforms with BLAT [33]. Based on the BLAT result, each EST was assigned to the best-matching isoform provided that the EST sequence was at least 100 bp long and the alignment region had to achieve 95% identity or higher. If more than one best-match existed, an arbitrary assignment was made. An expression profile of the representative isoforms was generated basing on EST assignments.

## Authors' contributions

WL and TC conceived and designed the experiments. TC and TW performed the experiments and analysed the data. TC wrote the manuscript. WL and WN supervised and revised the manuscript.

## Competing interests

The authors declare that they have no competing interests.

## Supplementary Material

Additional file 1 **Box plot of protein lengths** Box plot of protein lengths for four groups of genes: A_F, N_F, A_S, and N_S (AS gene families genes, no AS gene families genes, AS singletons, no AS singletons, respectively) of mouse across three different identity criteria (>10, >50, >90). This pattern of length distribution is similar to the pattern observed for human. Click here for file

Additional file 2 **Number of domain(s) distribution** Number of domain(s) for genes of four groups of genes: A_F, N_F, A_S, and N_S (AS gene families genes, no AS gene families genes, AS singletons, no AS singletons, respectively) identified with identity criteria >50 and >90. Click here for file

Additional file 3 **Average EST hits per transcript for gene families and singletons with/without AS.** The labels on the x axis are A_F, N_F, A_S, and N_S (for AS gene families genes, no-AS gene families genes, AS singletons, no AS singletons, respectively) across different identity criteria. Genes within N_F have relatively fewer EST hits compared to genes within A_F. These genes within N_F may actually have alternative splicing isoforms but are classified as no AS, resulting from the relatively lower expression level. We noticed that expression of these recently duplicated genes (gene families identified under identity criterion >90) are low compared to their comparable groups e.g. A_F (>90) compared to A_F (>10) and A_F (>50). Click here for file
