# Supplementary figures and images for: Interrogation of alternative splicing events in duplicated genes during evolution
Source: BMC Genomics. 2011 Nov 30;12(Suppl 3):S16. doi: 10.1186/1471-2164-12-S3-S16 (PMC3333175; doi:10.1186/1471-2164-12-S3-S16)

# Length distribution (mouse)

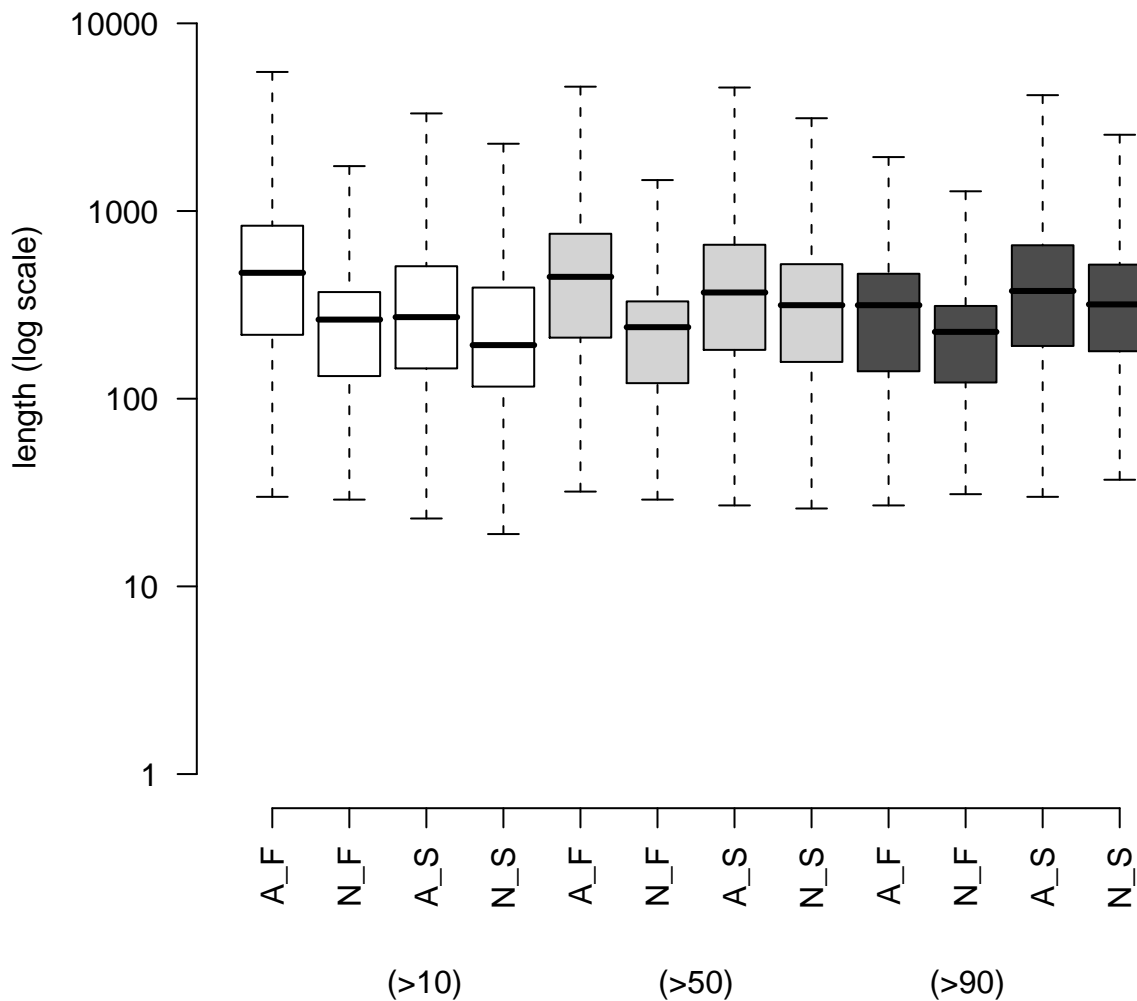

Supplement: Additional file 1 — Box plot of protein lengths Box plot of protein lengths for four groups of genes: A_F, N_F, A_S, and N_S (AS gene families genes, no AS gene families genes, AS singletons, no AS singletons, respectively) of mouse across three different identity criteria (>10, >50, >90). This pattern of length distribution is similar to the pattern observed for human. [file 1471-2164-12-S3-S16-S1.pdf]

**Average EST hits per transcript**

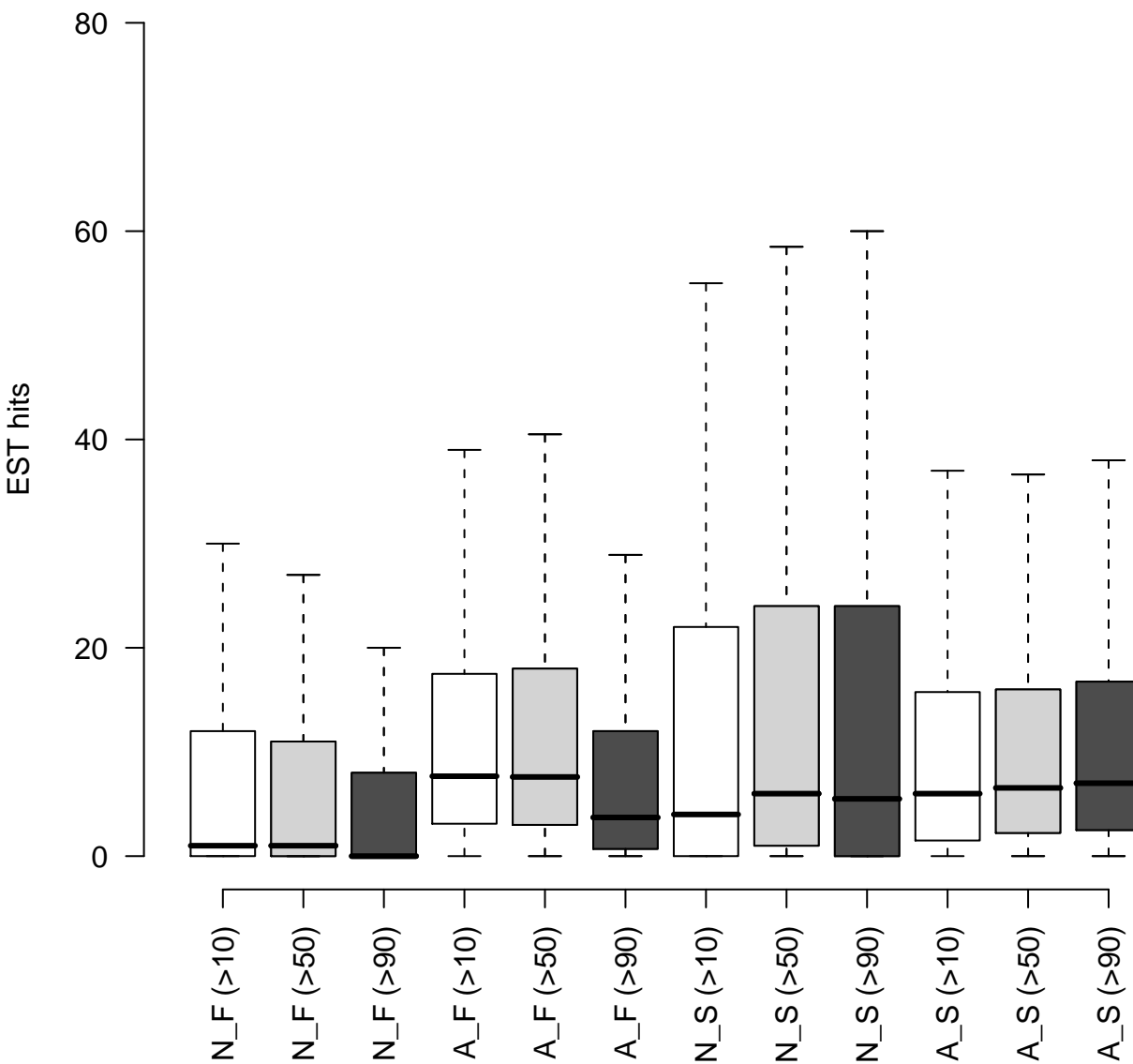

Supplement: Additional file 3 — Average EST hits per transcript for gene families and singletons with/without AS. The labels on the x axis are A_F, N_F, A_S, and N_S (for AS gene families genes, no-AS gene families genes, AS singletons, no AS singletons, respectively) across different identity criteria. Genes within N_F have relatively fewer EST hits compared to genes within A_F. These genes within N_F may actually have alternative splicing isoforms but are classified as no AS, resulting from the relatively lower expression level. We noticed that expression of these recently duplicated genes (gene families identified under identity criterion >90) are low compared to their comparable groups e.g. A_F (>90) compared to A_F (>10) and A_F (>50). [file 1471-2164-12-S3-S16-S3.pdf]
