# Supplementary material for: Interrogation of alternative splicing events in duplicated genes during evolution
Source: BMC Genomics. 2011 Nov 30;12(Suppl 3):S16. doi: 10.1186/1471-2164-12-S3-S16 (PMC3333175; doi:10.1186/1471-2164-12-S3-S16)
Supplement: Additional file 2 — Number of domain(s) distribution Number of domain(s) for genes of four groups of genes: A_F, N_F, A_S, and N_S (AS gene families genes, no AS gene families genes, AS singletons, no AS singletons, respectively) identified with identity criteria >50 and >90. [file 1471-2164-12-S3-S16-S2.pdf]

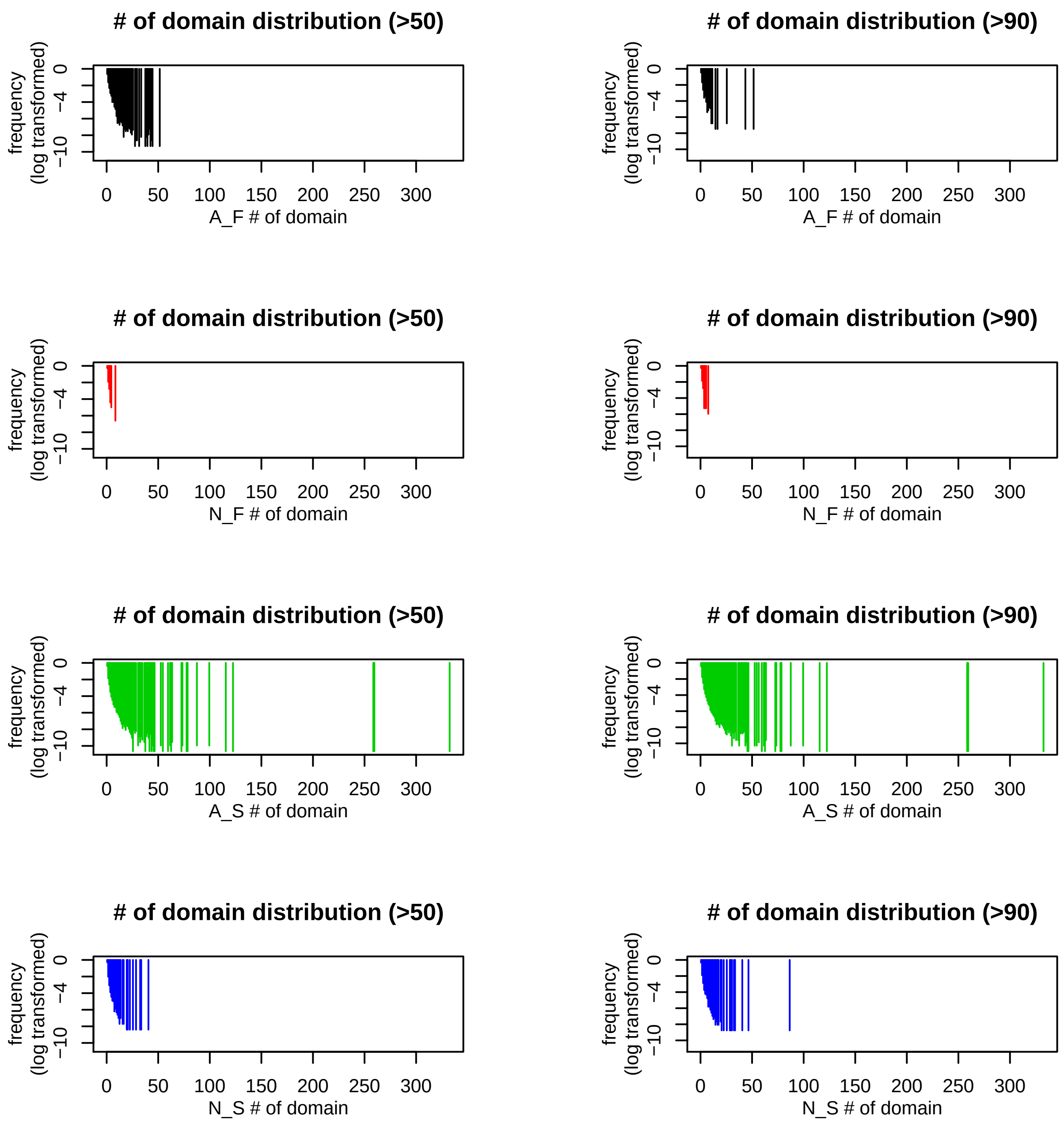

# of domain distribution (>50)
frequency
(log transformed)
0
−4
−10
0
50
100
150
200
250
300
A_F # of domain
# of domain distribution (>90)
frequency
(log transformed)
0
−4
−10
0
50
100
150
200
250
300
A_F # of domain
# of domain distribution (>50)
frequency
(log transformed)
N_F # of domain
# of domain distribution (>90)
frequency
(log transformed)
N_F # of domain
# of domain distribution (>50)
frequency
(log transformed)
A_S # of domain
# of domain distribution (>90)
frequency
(log transformed)
A_S # of domain
# of domain distribution (>50)
frequency
(log transformed)
N_S # of domain
# of domain distribution (>90)
frequency
(log transformed)
N_S # of domain
